# Leaf variegation in a barley EMS mutant is controlled by two epistatic mutations

**DOI:** 10.1007/s00122-026-05370-y

**Published:** 2026-10-14

**Authors:** Hélène Pidon, Shobhashree Rajulareddy Nagireddy, Michael Melzer, Axel Himmelbach, Nils Stein

**Affiliations:** 1 Leibniz Institute of Plant Genetics and Crop Plant Research (IPK) Gatersleben, Seeland, Germany; 2 UMR AGAP Institut, Univ. Montpellier, CIRAD, INRAE, Institut Agro, Montpellier, France; 3 Institute of Agricultural and Nutritional Sciences, Martin Luther University Halle-Wittenberg, Halle, Germany

## Abstract

**Key message:**

**We identified digenic control of leaf variegation in a barley mutant. Candidate genes, identified through whole-genome resequencing, are consistent with possible roles in chloroplast proteostasis.**

**Abstract:**

Variegation mutants provide valuable insights into chloroplast biogenesis. We characterized a newly identified variegated barley mutant, in which the phenotype is controlled by two epistatic recessive genes. The causal loci, *var4* and *var5*, were mapped on chromosomes 2H and 3H. We used genotyping-by-sequencing, linkage mapping, and PACE genotyping, followed by whole-genome resequencing to identify candidate genes. Our two top candidate genes are an *NBR1-like selective autophagy receptor* gene and a *DNAJ-domain-containing* gene, respectively. Based on their homology-based functional annotation, both candidates could be implicated in chloroplast proteostasis, regulating protein import, folding, and/or degradation. While functional confirmation is still required, mild, independent defects in proteostasis from each mutation may act synergistically to surpass a functional threshold, impairing chloroplast development in early leaves while allowing partial recovery in later stages. These findings reveal a novel digenic basis for variegation in barley and identify candidate genes and pathways linked to chloroplast proteostasis.

**Supplementary Information:**

The online version contains supplementary material available at https://doi.org/10.1007/s00122-026-05370-y.

## Introduction

Since the 1950s, the induction of mutations in plant populations has been extensively used, either to obtain new traits or to study the genetic basis of a phenotypic defect to shed light on developmental processes. In this frame, the study of photosynthesis-related mutants exhibiting abnormal leaf coloration is of particular interest to study chloroplast development (Taylor et al. 1987; Barkan 1998; Leon et al. 1998).

Chloroplast biogenesis is the process by which chloroplasts differentiate from proplastids (Pogson and Albrecht 2011). This complex process relies on signaling between the nucleus and the chloroplasts and requires both plastid and nuclear genes. Out of approximately 3,000 chloroplast proteins, less than 100 are encoded by the plastome (Li and Chiu 2010; Petersen et al. 2013). Most mutations resulting in aberrant leaf coloration are, therefore, affecting nuclear genes, and their identification and functional characterization brings us one important step closer to the understanding of regulatory mechanisms of chloroplast biogenesis.

Among phenotypes with chlorophyll deficiency, leaf variegation is particularly interesting. It is characterized by areas of the leaf with a certain level of pigment deficiency, either in patches in dicotyledons or stripes in monocotyledons (Yu et al. 2007). Variegated mutants usually have well-developed chloroplasts in green leaf sectors and defective ones in the pigment-deficient sectors. An important advantage of variegated mutants over albinos (complete chlorophyll deficiency) is that they usually survive and produce seeds, which enables genetic studies. Somatic chimerism can be the reason for leaf variegation, where cells of each sector present a different genotype. However, in the case of mutants, the genotype is most likely identical in the whole plant.

Several variegation genes have been cloned and characterized through the years, both in *Arabidopsis thaliana* (Wu et al. 1999; Chen et al. 2000; Takechi et al. 2000; Sakamoto et al. 2002; Wang et al. 2004; Zheng et al. 2016; Zagari et al. 2017) and in other plant species such as rice (Hayashi-Tsugane et al. 2014; Li et al. 2018), barley (Li et al. 2019; Overlander-Chen et al. 2024), or tomato (Song et al. 2023; Dechkrong et al. 2024). The best-described genes are *Arabidopsis thaliana VAR1* and *VAR2*, encoding proteins of the filamentation temperature-sensitive (FtsH) metalloprotease family: FtsH5 and FtsH2, respectively (Lindahl et al. 1996, 2000; Chen et al. 2000; Takechi et al. 2000; Sakamoto et al. 2002). Current models propose that chloroplast FtsH proteins assemble into hetero-oligomeric complexes composed of two types of subunits: the type A proteins FtsH1/FtsH5(VAR1) and the type B proteins FtsH2(VAR2)/FtsH8 (Yu et al. 2004). In the case of VAR2, a threshold model explains variegation, whereby FtsH8 partially compensates for the loss of functional FtsH2/VAR2 in *var2* mutants. In barley, three variegation mutations were studied in greater detail: *albostrians*, caused by a mutation in *Hordeum vulgare CCT Motif Family gene 7* (*HvCMF7*) (Li et al. 2019), *luteostrians*, for which the *ATP-Dependent Clp Protease Subunit C1* has been postulated as a convincing candidate gene (Li et al. 2021), and *grandpa1.a*, caused by a large deletion in a gene coding for plastid terminal oxidase homologous to *A. thaliana IMMUTANS* (Overlander-Chen et al. 2024). More barley variegated mutants are listed in the NordGen database (Hansson et al. 2024; https://bgs.nordgen.org/), such as *var1–var3* (Variegated 1–3), *wst1–wst7* (White streak 1–7), and *yst1–yst5* (Yellow streak 1–5).

Here, we studied the genetic basis of a leaf variegation phenotype induced by the alkylating agent ethyl methane sulfonate (EMS) in the genetic background of the two-rowed winter barley cultivar Igri. We showed that, unlike previously identified variegation traits, variegation is here controlled by two epistatic genes, which we named Variegated 4 (*var4*) and Variegated 5 (*var5*), respectively, in accordance with the naming conventions of barley genes (Hansson et al. 2024). Performing whole-genome sequencing of variegated and green progenies of the mutant x wild-type backcross, we narrowed down a small number of candidate genes for the two loci, which hint to chloroplast proteostasis as the developmental process that is centrally affected by the mutation.

## Materials and methods

### Plant material

The variegated parent is a fourth-generation selfed (M4) mutant from an EMS-mutagenized population of the barley cultivar Igri, obtained according to the protocol of Gottwald et al. (2009) with 40 mM of EMS treatment for 16 h. Three F_2_ populations were constructed: The cultivar Alraune was used as wild-type (WT) in reciprocal crosses to establish the populations AM (Alraune x mutant) and MA (mutant x Alraune), respectively. Reciprocal crosses were initiated to test for nucleus- or cytoplasm-controlled variegation. In addition, the true WT cultivar Igri was used for backcrossing to form the IM (Igri x mutant) population. F_3_ progenies of four and two variegated F_2_ from the AM and MA populations, respectively, were grown to confirm the segregation.

All plants were cultivated in a greenhouse under a day/night temperature cycle of 20 °C/15 °C and a photoperiod of 16 h of light and 8 h of darkness. Natural light was extended when necessary using incandescent lamps (SON-T Agro 400, MASSIVEGROW) at 300 μmol photons m^–2^ s^–1^.

### Phenotyping and Ultrastructural Analysis

The presence of stripes on the leaves was assessed as a qualitative trait 10 days–3 months after sowing, such that a single variegated leaf was sufficient to classify the plant as variegated. Leaves of the mutant and WT Igri plants were collected 14 days and 6 months after sowing and prepared for morphological and ultrastructural analysis. For this purpose, cuttings of 1–2 mm^2^ of the central part of the leaves of at least five plants were fixed with glutaraldehyde and paraformaldehyde, embedded in resin, sectioned, and examined by light and transmission electron microscopy (TEM) according to the protocol of (Li et al. 2021).

### Genotyping-by-sequencing

To perform linkage mapping, 269 and 183 F_3_ plants from AM and MA populations, respectively, were genotyped using genotyping-by-sequencing (GBS). Genomic DNA was extracted according to the guanidine isothiocyanate-based protocol described by Milner et al. (2019). GBS library preparation using *PstI* and *MspI* enzymes and sequencing followed essentially a previously described procedure (Zhang et al. 2024). Sequencing (single-read, SP flow cell with 100-cycle kit) was performed using a custom sequencing primer (Wendler et al., 2014) and the Illumina NovaSeq6000 device (Illumina, Inc., San Diego, CA, USA) at IPK-Gatersleben.

The GBS sequencing data were aligned to Morex reference genome version 3 (Mascher et al. 2021), and variants were called as described by Milner et al. (2019). The variants table was filtered as followed: homozygous and heterozygous variant calls with a depth of sequencing inferior to 4 and 6, respectively, were filtered out, variant sites were accepted wherever they had a minimum mapping quality score of 40, a maximum fraction of 20% of missing data, a maximum of 60% of heterozygous call, and a minimum allele frequency of 30%. GBS data from the parents were used to assign each call to its respective parental allele.

### Linkage mapping

The genotype matrix obtained from GBS was additionally filtered to construct the linkage map. Only variant sites with less than 15% missing data and individuals with less than 25% missing data were retained. The data were manually curated to remove suspicious double crossing overs.

The linkage map was built in R 4.1.3 with the package ASMap (Taylor and Butler 2017), using the mstmap.cross function with the chromosome information from the GBS mapping as linkage group and a high *p*-value of 0.01 to avoid splitting them into several linkage groups, as well as the option “anchor=TRUE” to respect the order of the markers on the chromosomes. The linkage map was visualized with the package LinkageMapView (Ouellette et al. 2018) and edited in Inkscape 1.1.2. Finally, the linkage mapping of the variegation trait was performed with the R/qtl package (Broman et al. 2003) using simple maximum likelihood-based interval mapping, performed via the EM algorithm and with a binary phenotype model, as well as composite interval mapping. The significance threshold was calculated based on 1000 permutations at a 5% alpha level, and the interval was determined based on 1-LOD drop (Lander and Botstein 1989).

### PACE genotyping

For a finer delimitation of the mapping intervals, polymorphisms identified in the candidate intervals in the GBS data from the parental lines, Alraune and the mutant, were developed into PCR Allele Competitive Extension (PACE) markers through 3CR Bioscience (Essex, UK) free assay design service (Supplementary Table S1). Genotyping with the PACE markers was carried out as described by Pidon et al. (2020) on 89 selected F_2_ plants, of which 21 were variegated, from AM and MA populations, and the parents of the populations.

### Whole-Genome Shotgun Sequencing and polymorphisms analysis

A total of 30 F_2_ individuals from the IM population, of which six exhibited a striped phenotype, were selected for whole-genome shotgun sequencing (WGS). DNA was extracted from young leaf tissue using the Milner et al. (2019) protocol. Libraries were prepared with Illumina Nextera DNA Flex Kit following the standard protocols from the manufacturer (Illumina, Inc., San Diego, CA, USA), quantified by qPCR (Mascher et al. 2013), and sequenced on the NovaSeq6000 platform using the XP workflow (S1 flow cell, paired-end, 2 × 151 cycles). High-quality reads were then mapped against two barley genomes: the reference genome Morex (Mascher et al. 2021) and the WT parent Igri (Jayakodi et al. 2024) as described above for GBS data.

Intervals of interest on the Igri genome were identified by blasting the flanking 100 bp of each Morex interval. SNPs located on chromosomal regions of interest were extracted using VCFtools (Danecek et al. 2011). Annotation of polymorphisms was carried out using SnpEff (Cingolani et al. 2012), employing the respective genome annotations. The variants were then filtered as follows: only SNPs with a single alternate allele were retained; among the six striped individuals, none could be fixed for the reference allele, and they had to include no more than one heterozygous genotype and at least two homozygous alternate genotypes; among the 24 non-striped individuals, at least one had to be homozygous for the reference allele and at least one homozygous for the alternate allele. Genes were defined as candidates if they displayed a filtered SNPs with “MODERATE” or “HIGH” predicted effects on protein, and were not classified as low confidence (LC) in the Morex genome, annotate as transposable elements, or had significant similarity to transposons based on BLAST searches.

The homologous genes in *Arabidopsis thaliana* were retrieved based on the TAIR annotation displayed in PanBARLEX (https://panbarlex.ipk-gatersleben.de/; Jayakodi et al. 2024). Orthologs in other monocotyledonous species were retrieved by identifying the orthogroups to which the genes belong in Monocots PLAZA 5.0 (Van Bel et al. 2022). The orthologs were then aligned, and the conservation of the amino acid mutated in the EMS mutant was assessed. The sequence logos in the 101 bp interval around these polymorphisms were generated using WebLogo 3 (Crooks et al. 2004).

## Results

### Description and segregation of the variegated phenotype

**Fig. 1:**
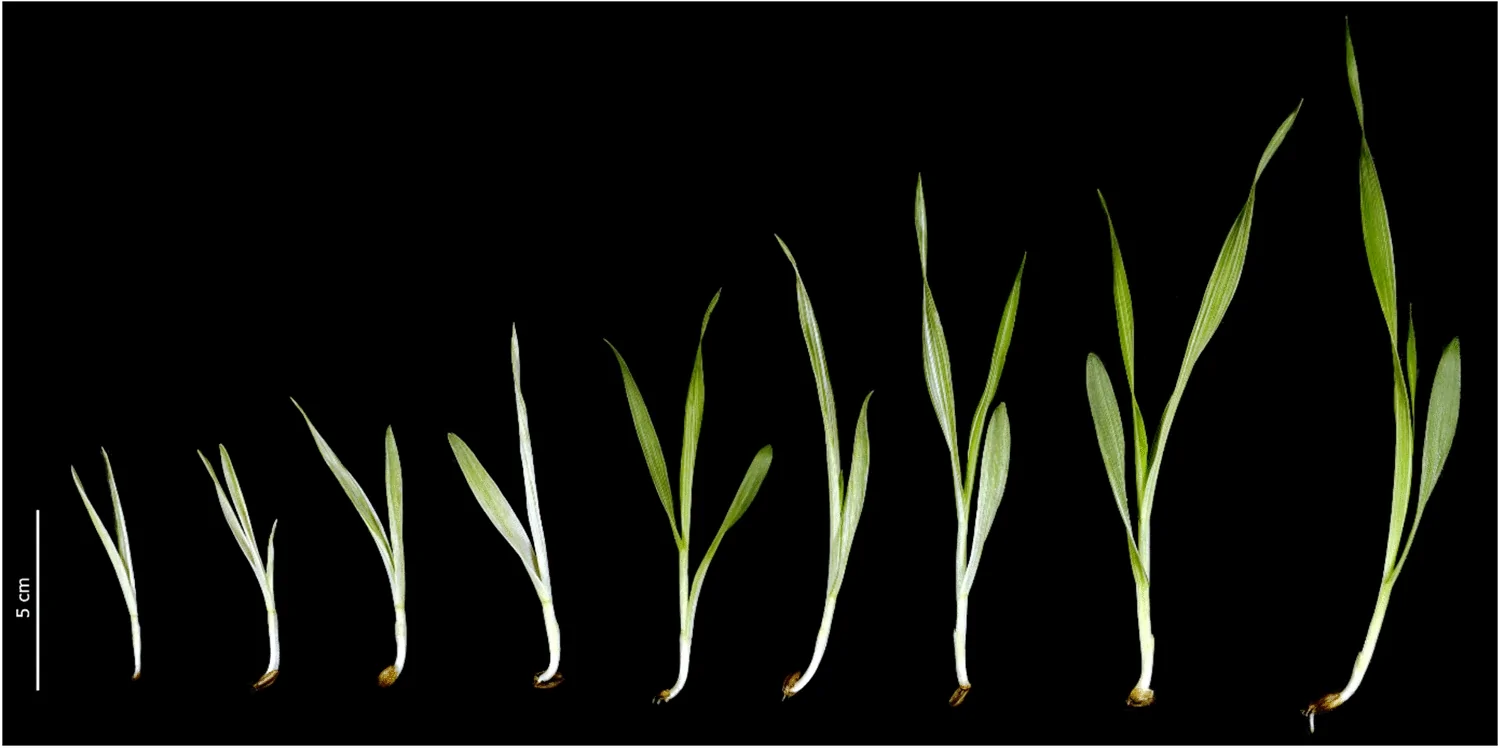
Penetration of the mutant phenotype on 14-day-old mutant plants. The mutant plantlets displayed diverse levels of variegation, from almost albino plantlets to plantlets with only a few white stripes. The level of penetration of the phenotype also affects growth

**Fig. 2:**
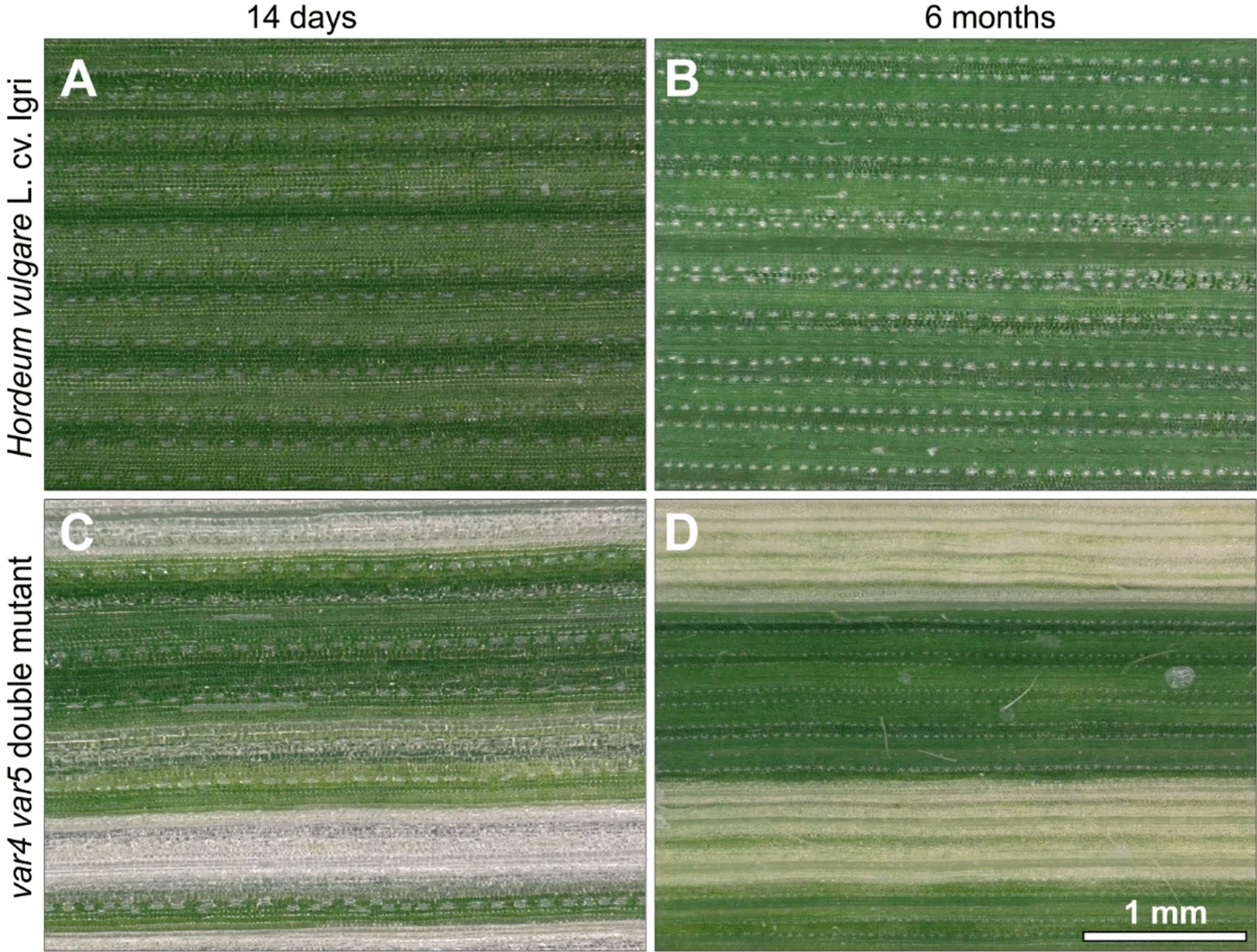
Digital microscopy observation of the central part of leaves from WT and mutant Igri plants 14 days (**A**, **C**) and 6 months (**B**, **D**) after sowing. Stripes are composed of cells from an identical cell line. After 6 months, the stripes are turning yellow, suggesting a partial recovery

The mutant plants display a white-striped variegated phenotype with different levels of penetration, from a mostly white plant to a low number of narrow white stripes (Fig. 1). As the plant grows, tillers present a different level of variegation. After 6 months of cultivation, the stripes are progressively turning yellow (Fig. 2). The F_3_ progenies of variegated F_2_ plants showed a similar but not as pronounced phenotype as the most variegated mutant plants. On rare instances, stripes did not occur on the primary tiller, but rather only on secondary ones.

To assess if there is a maternal inheritance of the variegation, the proportion of variegated plants in the reciprocal crosses AM and MA was compared. These proportions were 17 out of 298 and 6 out of 203 (Table 1, Supplementary Table S2), respectively, and, thus, are not statistically different (Chi-square test of independence, *χ*^2^(1, *N* = 501) = 2.083, *p *= 0.149), indicating that the trait is nuclear-encoded and inherited. While a minor maternal or cytoplasmic effect cannot be completely excluded, the similarity of segregation patterns between reciprocal crosses argues against a major contribution of such effects. The two populations are, therefore, treated as one for the rest of the study.

**Table 1:** Plant phenotypes in the three F_2_ populations

Population	Variegated	Wild type	*χ*^2^ goodness of fit 1:15	*p*-value
AM	17	281	0.151	0.697
MA	6	197	3.760	0.052
IM	6	89	0.001	0.979

The number of variegated and wild-type plants are indicated. The Chi-square test of goodness of fit for the 1:15 ratio and the corresponding *p*-value in each population have been calculated

The segregation of the variegation trait in the combined AM-MA population fit the segregation ratio of 1:15, which could be expected for two recessive epistatic factors (Chi-square goodness of fit test *χ*^2^(1) = 2.354, *p *= 0.125). The segregation in the IM population (Table 1, Supplementary Table S2) does not statistically differ from the one in the AM-MA population (Chi-square test of independence, *χ*^2^(1, N = 596)=0.513, *p *= 0.474), confirming that both genetic factors are inherited from the EMS mutant. The segregation of variegation in F_3_ was checked on the progenies of six variegated F_2_. All 12 F_3_ plants per line were variegated, confirming that the genotype is fixed at the responsible loci in variegated plants.

### Genetic mapping reveals two epistatic loci

**Fig. 3:**
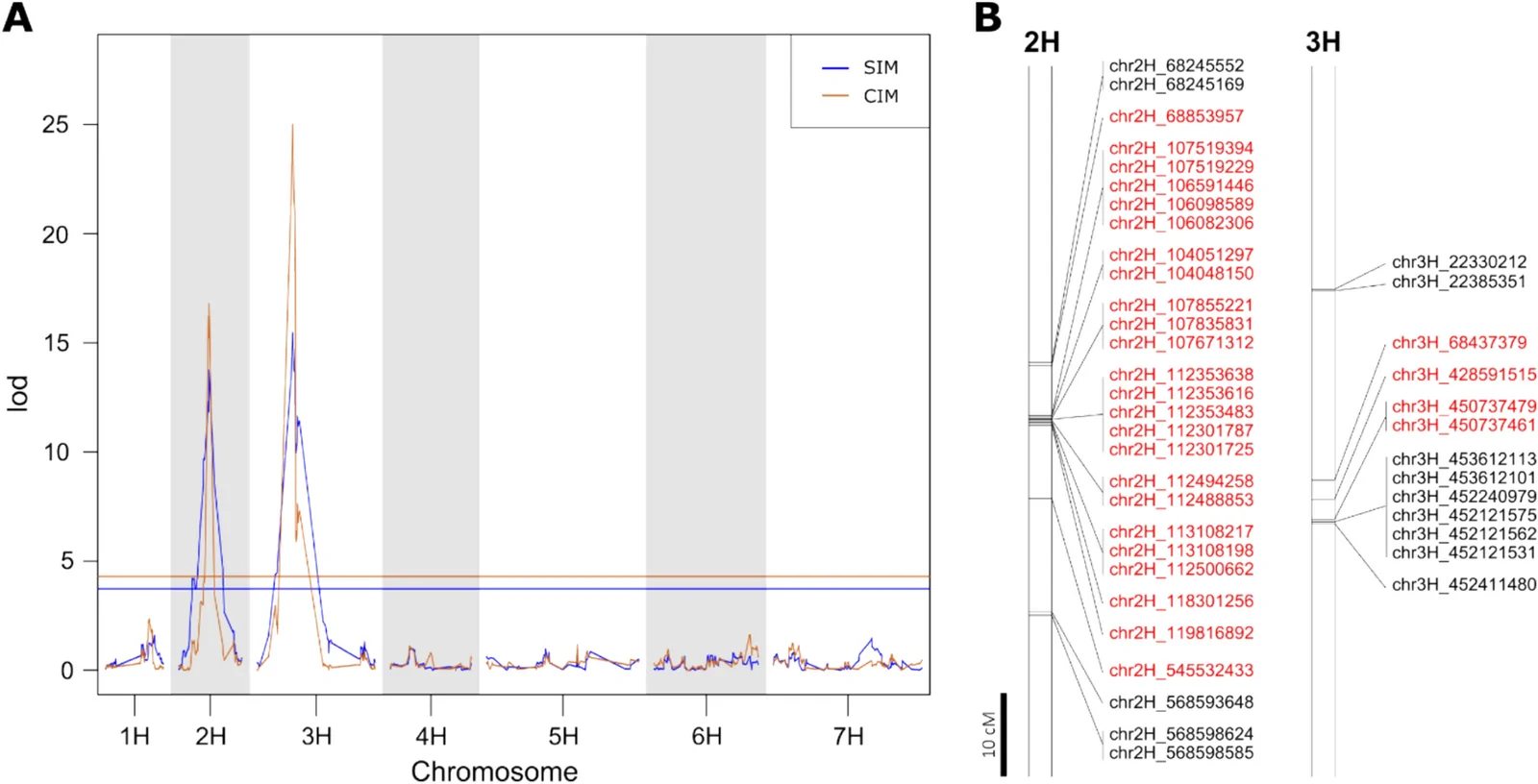
Interval mapping of the variegation trait. **A.** LOD scores calculated with simple interval mapping (SIM, blue) and composite interval mapping (CIM, orange) along the chromosomes. The horizontal bars represent the 5% probability thresholds calculated based on 1000 permutations. **B.** Graphical representation of the significant intervals. The markers in red represent the intervals calculated by 1-LOD drop

After filtering of the GBS data, 265 and 181 plants from the AM and MA populations, respectively, and 3,022 SNPs remained (Supplementary Table S3). Simple interval mapping and composite interval mapping, performed on the combined AM and MA populations, identified two major loci, confirming the hypothesis derived from the segregation pattern (Fig. 3A, Supplementary Table S4). Those two loci, located on chromosome 2H and chromosome 3H, were named Variegated 4 (gene symbol: *var4*) and Variegated 5 (*var5*), respectively. The respective recessive mutant alleles are *var4.a* and *var5.a.* Based on a 1-LOD drop applied to the composite interval mapping model, the interval of *var4* covers 476.7 Mbp from markers chr2H_68853957 and chr2H_545532433. The interval of *var5* spanned over 382.3 Mbp and contains markers chr3H_68437379, chr3H_428591515, chr3H_450737479, and chr3H_450737461 (Fig. 3B).

**Fig. 4:**
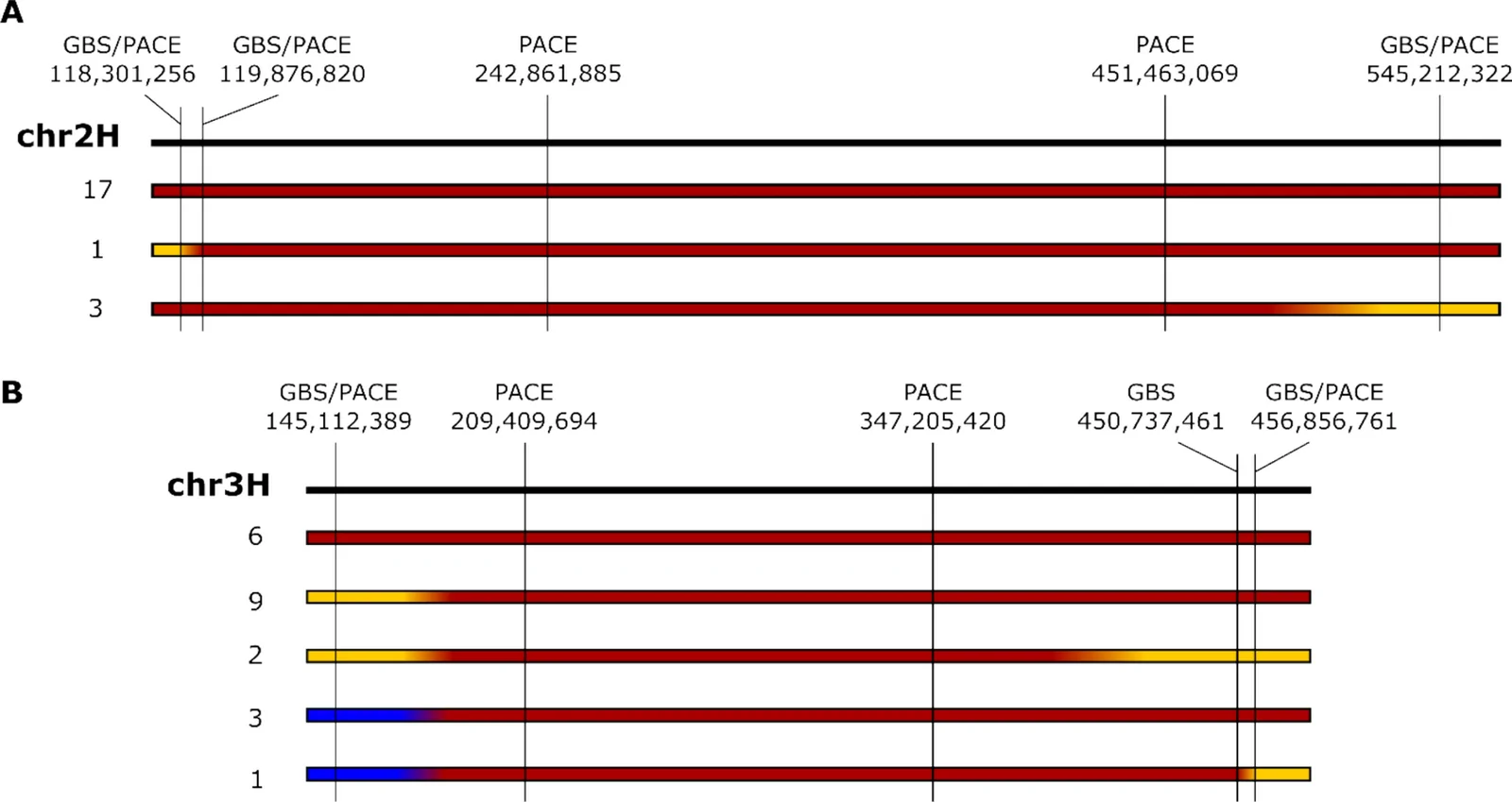
Graphical representation of the genotype at *var4* (**A**) and *var5* (**B**) loci of the 21 variegated F_2_ plants. Vertical bars represent the position of the markers, identified by their nature (GBS, PACE, or both) and their physical position on the Morex reference genome. Each horizontal rectangle represents a genotype in the interval. Red, blue, and yellow sections depict homozygous mutant, homozygous Alraune, and heterozygous alleles, respectively. The number on the left side of the bar shows the number of variegated F_2_ plants displaying this genotype

A visual inspection of the genotype and crossover matrix confirmed and refined the intervals to chr2H: bp-position 118,301,256—545,212,322 and chr3H: bp-position 145,112,389—450,737,461 intervals for *var4* and *var5*, respectively. Only plants homozygous for the mutant allele at both loci presented the variegated phenotype. Genotyping with PACE markers of 89 F_2_ plants confirmed those intervals, without allowing to further reduce them (Fig. 4, Supplementary Table S5).

The mapping intervals of *var4* and *var5* mutations are annotated with 5,151 and 3,409 genes, including 3,928 and 2,577 protein-coding genes, of which 2,071 and 1,279 are annotated with high confidence, respectively.

### Five genes are candidates in each interval

To reduce the number of candidate genes, we performed WGS on 30 F_2_ plants from the backcross between the mutant and the WT cultivar Igri (IM population) and compared the polymorphisms observed on the six variegated plants and 24 green ones and their effects on coding genes (Supplementary Tables S6, S7, S8, and S9). The same list of five candidate mutations on each interval was found after mapping the WGS data on Morex or Igri genomes and filtering the results for their impact on protein-coding genes annotated with high confidence (Table 2).

**Table 2:** Candidate genes and their mutation in the *var4 var5* double mutant

Morex gene ID	Igri gene ID	Pol. coordinates Morex	Pol. coordinates Igri	Nucl Pol	Typical EMS?	Proteic Pol	Functional annotation	*Arabidopsis thaliana* homolog
HORVU.MOREX.PROJ.2HG00123910	HORVU.IGRI.PROJ.2HG00125410	chr2H:206,718,858	chr2H:205,712,373	C>T	yes	A18V	Tetratricopeptide repeat (TPR)-like superfamily protein	AT5G46400 / PRP39-2
HORVU.MOREX.PROJ.2HG00125010	HORVU.IGRI.PROJ.2HG00126500	chr2H:216,446,602	chr2H:215,412,966	T>A	no	K72*	tRNA wybutosine-synthesizing protein 2 homolog	AT4G04670 / TRM5C
HORVU.MOREX.PROJ.2HG00727300	HORVU.IGRI.PROJ.2HG00707640	chr2H:369,405,874	chr2H:366,262,896	G>A	yes	T65I	Unknown protein	-
HORVU.MOREX.PROJ.2HG00150700	HORVU.IGRI.PROJ.2HG00151540	chr2H:499,124,413	chr2H:494,235,019	C>T	yes	R219K	Ribonuclease H-like superfamily protein	AT3G25270
HORVU.MOREX.PROJ.2HG00154410	HORVU.IGRI.PROJ.2HG00155310	chr2H:525,960,629	chr2H:521,187,072	C>T	yes	G410D	ZZ-type zinc finger-containing protein	AT4G24690 / NBR1
HORVU.MOREX.PROJ.3HG00226420	HORVU.IGRI.PROJ.3HG00227510	chr3H:185,882,934	chr3H:187,024,402	C>T	yes	R63C	phytochrome kinase substrate 4	AT5G04190 / PSK4
HORVU.MOREX.PROJ.3HG00238870	HORVU.IGRI.PROJ.3HG00239830	chr3H:345,372,133	chr3H:343,527,666	G>A	yes	S696F	DNAJ heat shock N-terminal domain-containing protein-like	AT5G18750
HORVU.MOREX.PROJ.3HG00239080	HORVU.IGRI.PROJ.3HG00240020	chr3H:347,359,958	chr3H:345,512,207	A>T	no	K38N	F-box/FBD/LRR protein	AT1G69630
HORVU.MOREX.PROJ.3HG00244660	HORVU.IGRI.PROJ.3HG00245600	chr3H:404,811,167	chr3H:402,278,225	C>T	yes	T1017I	Histone-lysine N-methyltransferase	AT4G27910 / SDG16
HORVU.MOREX.PROJ.3HG00246700	HORVU.IGRI.PROJ.3HG00247670	chr3H:422,167,470	chr3H:419,628,399	A>G	no	W119R	Lectin-domain-containing receptor kinase A4.3	AT1G66235

The coordinates of the mutation are given in relation to Morex v3 (Mascher et al. 2021) and Igri (Jayakodi et al. 2024) genome assemblies. The functional annotation and *A. thaliana* homolog provided are those displayed in the PanBARLEX portal (https://panbarlex.ipk-gatersleben.de/)

Among the five candidate mutations for *var4.a*, four are typical EMS mutations (C to T or G to A) leading to amino acid substitutions, and one is a non-typical EMS mutation (T to A) causing a premature stop in a protein annotated as tRNA wybutosine-synthesizing. Moreover, three out of five are heterozygous in one of the six striped plants (MZ42_46_3_36), and there is no coverage at *HORVU.MOREX.PROJ.2HG00727300* locus, annotated as an unknown protein. Only the polymorphism in *HORVU.MOREX.PROJ.2HG00154410* is homozygous for the alternate allele in all six striped plants and, therefore, the best candidate. This gene is one of the two closest homologs of *A. thaliana NBR1* which codes for an ubiquitin-binding autophagy receptor protein directing the autophagy of damaged chloroplasts as well as regulating chloroplast protein import through the control of translocon at the outer envelope membrane of chloroplasts (TOC) protein levels (Lee et al. 2023; Wan et al. 2023). In the *var4 var5* double mutant, the mutation replaces a glycine with an aspartic acid in the *NBR1* homolog. This residue lies just 16 amino acids upstream of the zinc finger domain, within the linker region that positions the domain for binding. The introduction of a negatively charged residue in this position could alter the domain’s orientation or electrostatic environment, potentially reducing interaction efficiency.

All candidate mutations for *var5.a* are responsible for amino acid changes and are homozygous in all six striped plants. Three of them have the typical EMS signature (in *HORVU.MOREX.PROJ.3HG00226420*, *HORVU.MOREX.PROJ.3HG00238870*, and *HORVU.MOREX.PROJ.3HG00244660*), and are, therefore, better candidates than the two that do not (in *HORVU.MOREX.PROJ.3HG00239080* and *HORVU.MOREX.PROJ.3HG00246700*). *HORVU.MOREX.PROJ.3HG00226420* is annotated as a phytochrome kinase substrate 4 and is homologous to *A. thaliana PKS4*, involved in phototropism and the regulation of light signaling (Schepens et al. 2008; Demarsy et al. 2012). The protein produced by *HORVU.MOREX.PROJ.3HG00244660* is a histone-lysine N-methyltransferase homologous to AtSDG16, involved in transcriptional regulation via chromatin modification, in particular in response to drought stress (Schuettengruber et al. 2011; Chen et al. 2017; Liu et al. 2018). Finally, *HORVU.MOREX.PROJ.3HG00238870* encodes a chaperone protein, DNAJ. DNAJ proteins are co-chaperones of HSP70s, ensuring correct folding of proteins and targeting misfolded proteins for degradation in collaboration with Clp proteases (Pulido et al. 2013; Pulido and Leister 2018; Rodriguez-Concepcion et al. 2019). While the amino acids affected by mutations in the other two candidates are poorly conserved, the substitution in HORVU.MOREX.PROJ.3HG00238870 affects a highly conserved serine—present in 284 out of 326 proteins in the orthogroup (Supplementary Fig. S1)—replacing it with phenylalanine in one of the protein’s two DUF3444 domains. This radical change in a domain that is highly conserved across most DNAJ proteins may impair its function in the described mutant.

Beyond these polymorphisms, EMS-type mutations were identified in non-coding regions. After mapping the WGS data to the Igri genome and applying an additional filter to retain only homozygous calls in striped plants, 402 and 505 mutations were detected at the *var4* and *var5* loci, respectively (Supplementary Tables 6, 7, 8, and 9). Among these, 15 occur in upstream gene regions at each locus. While these may include polymorphisms in regulatory elements and cannot be ruled out as the underlying cause of the phenotype, they do not appear to impact obvious candidate genes.

### Chloroplast ultrastructure in the *var4 var5* double mutant

**Fig. 5:**
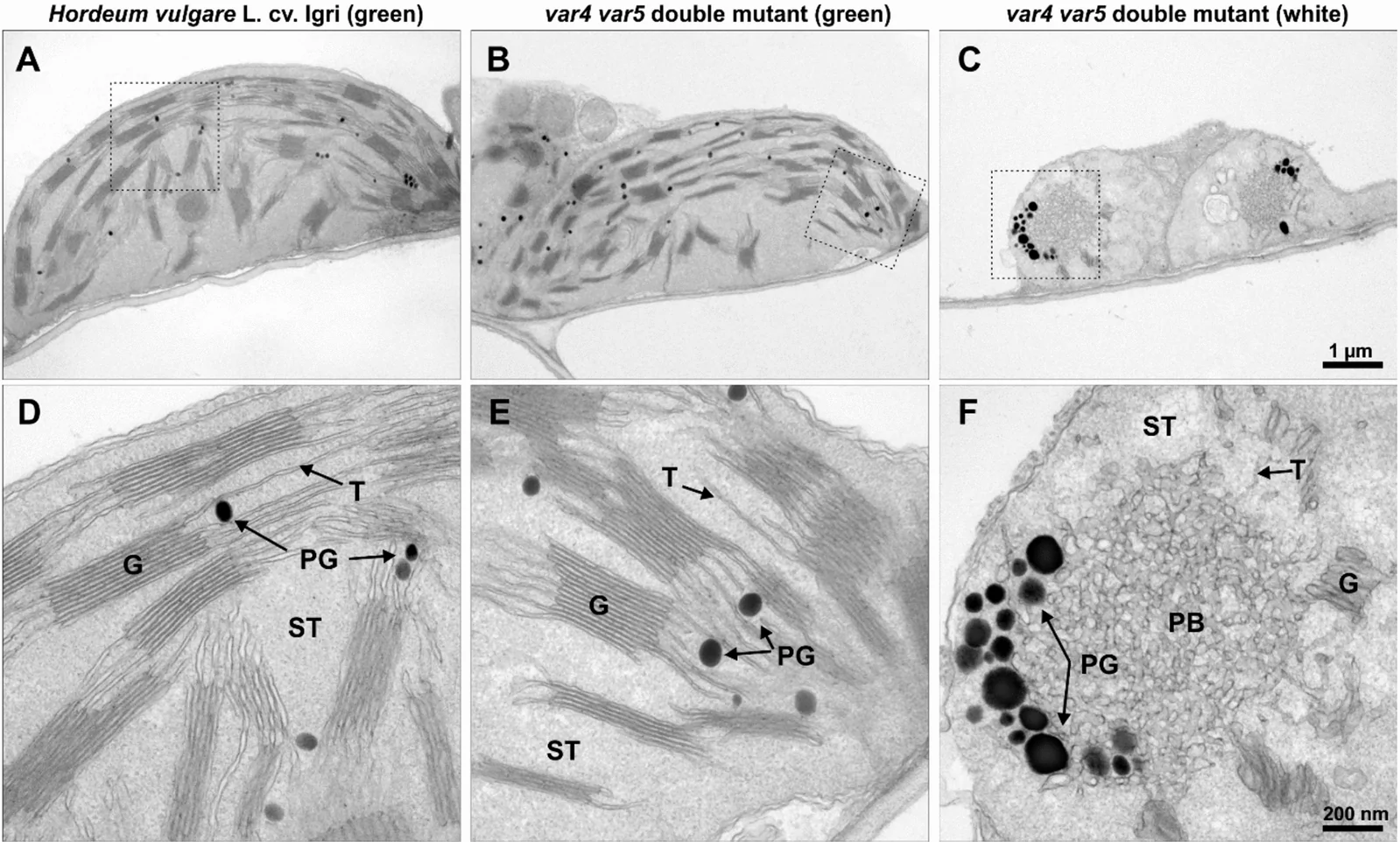
Chloroplast ultrastructure of WT Igri and *var4 var5* double mutant plants 14 days after sowing. Chloroplast with close up of WT Igri **(A, D)**, green **(B, E),** and white leaf sectors of the *var4 var5* double mutant **(C, F)**, respectively. G, grana; PB, prolamellar body, PG, plastoglobuli; ST, stroma thylakoid; and T, thylakoid

Chloroplasts of leaf samples of 14-day-old (white stripes in the mutant) and 6-month-old WT and mutant plants after sowing (stripes turned yellow) were analyzed by TEM. Chloroplasts in the WT and green sectors of the mutant leaves display well-developed stroma and grana thylakoids (Fig. 5 A, B, D, E). In contrast, chloroplasts of the white sectors of 14-day-old mutant plants appear significantly smaller. In addition, remaining prolamellar bodies, poorly developed thylakoids, and a lack of grana stacks indicate impaired chloroplast biogenesis (Fig. 5C, F).

**Fig. 6:**
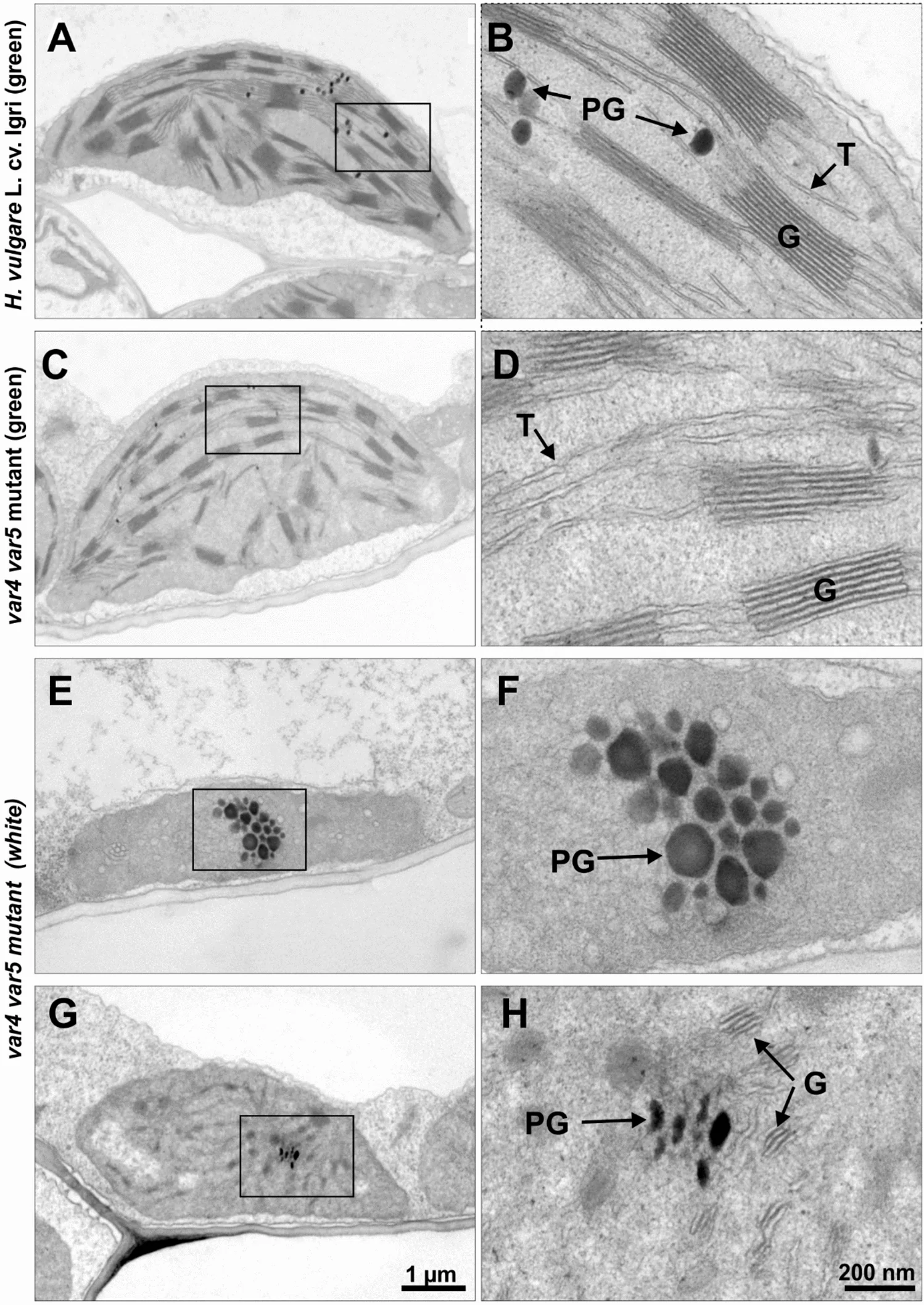
Chloroplast ultrastructure of WT Igri plant and *var4 var5* double mutant 6 months after sowing. Chloroplast with close up of Igri **(A, B)**, green **(C, D),** and yellow sectors of *var4 var5* double mutant **(E, F, G, H)**, respectively. G, grana; ST, stroma thylakoid; PB, prolamellar body; and PG, plastoglobuli

After 6 months of growth, chloroplasts of WT plants and green sectors of mutant variegated plants appear fully developed (Fig. 6A, B, E, F). However, in the now yellow sectors of the mutant, different levels of chloroplast development were observed, from chloroplasts with similar disorganization as observed after 14 days (Fig. 6C, G), up to chloroplasts with intermediate size and characterized by thylakoids and small grana (Fig. 6D, H). This observation suggests that chloroplast development is limited or rests at a certain level in the variegated segments of mutant leaves during plant growth.

## Discussion

Identification of the genetic basis of variegation is an important tool for a better understanding of chloroplast biogenesis. Here, we studied a newly identified variegated barley mutant in which we showed that variegation is controlled by duplicate dominant epistasis, the WT alleles being dominant. To our knowledge, this is the first time that a digenic control has been identified for chloroplast-deficient mutants. However, pairs of genes have been identified as playing a role in variegation, such as *VAR2*/*FtsH8,* which code for proteins part of the same oligomeric complex (Yu et al. 2004). The phenotype caused by *var2* is also enhanced by other chloroplastic genes that interact with it (Wang et al. 2018; Qi et al. 2020). The genetic control of the mutant variegation could be explained by an additive effect of the two mutations combined with a threshold-dependent mechanism similar to the one described for the *Arabidopsis* locus *Var2* (Takechi et al. 2000; Yu et al. 2007) and the barley mutant *albostrians* (Li et al. 2019).

A distinctive feature of the reported mutant here is that the initially white stripes of the early developmental stage turn visibly yellow after about 6 months under greenhouse conditions. Ultrastructural analysis of the chloroplasts in the white sectors at early developmental stages shows prolamellar bodies, barely developed thylakoids, and grana. The chloroplast development in those cells appears to be clearly disturbed. After 6 months, a proportion of the plastids in cells of the yellow sectors have better-formed thylakoids and small grana, although these are much less developed than in the green leaf sectors of the mutant or in the WT. These observations suggest a partial recovery of the chloroplast biogenesis process at a later plant developmental stage, possibly caused by the expression of other genes or a shift in the postulated activity threshold level.

The two new genes, *var4* and *var5*, were mapped with low resolution on chromosomes 2H and 3H, in 426.1 and 306.6 Mbp intervals, respectively. These intervals do not overlap with the ones described for other variegation loci in barley (Li et al. 2019, 2021; Overlander-Chen et al. 2024). Using WGS of variegated and green progenies of the mutant backcrossed with the WT, we were able to identify five candidate genes for each of the two intervals on the basis of their sequence variant profile. Interestingly, only one of the candidate mutations is a loss of function, while all the others are amino acid substitutions, with milder effects expected. This could account for the unusual detection of two loci in epistasis, as their mild effects may only pass a threshold and produce a visible phenotype when combined.

Because of the digenic recessive inheritance of the trait, the number of fully informative individuals is limited. While this would represent a major constraint in a classical map-based cloning framework, mutation mapping approaches partially overcome this limitation by integrating recombination information with the identification of induced variants (Abe et al. 2012). In such approaches, the search space is constrained by the limited number of EMS-induced mutations, and candidate genes are prioritized based on both their sequence characteristics and segregation patterns. This reduces the reliance on large numbers of recombinants and enables candidate identification within relatively broad mapping intervals.

At *var4* locus, *HORVU.MOREX.PROJ.2HG00154410* emerges as a strong candidate gene. It is homologous to *A. thaliana* *AT4G24690*, which encodes NBR1, a selective autophagy receptor and adaptor. NBR1 has been shown to recognize and mediate the degradation of photodamaged chloroplasts via a microautophagy-like process, thereby preventing the accumulation of dysfunctional organelles under high light conditions (Lee et al. 2023). In this pathway, the ubiquitin-binding UBA2 domain of NBR1 interacts with the ubiquitinated surface of photodamaged chloroplasts and promotes their delivery to the vacuole for degradation. NBR1 also contributes to chloroplast protein homeostasis (proteostasis) by regulating the abundance of TOC components (Wan et al. 2023). Changes in TOC protein levels affect the rate of protein import into chloroplasts and, consequently, the capacity of these organelles to adapt to different environmental conditions such as heat stress and UV-B radiation. In our barley mutant, alteration of NBR1 function could impair the clearance of damaged chloroplasts—leading to the accumulation of aberrantly developed organelles in specific cell types—or disrupt the adjustment of protein import in response to environmental conditions, thereby compromising chloroplast proteostasis.

At the *var5* locus*,* *HORVU.MOREX.PROJ.3HG00238870* emerges as a promising candidate. This gene encodes a DNAJ-domain protein, a member of a co-chaperone family that functions as a specificity factor for Hsp70 chaperones (Pulido and Leister 2018). DnaJ proteins recognize unfolded or misfolded substrates and deliver them to Hsp70s, which then mediate either their refolding or degradation. In chloroplasts, numerous DnaJ proteins have specialized roles in protein import, folding, assembly, and repair of essential photosynthetic and metabolic complexes. A well-characterized example is AtJ20, a plastid J-protein that specifically binds misfolded deoxyxylulose 5-phosphate synthase (DXS), an aggregation-prone enzyme, and delivers it to chloroplastic HSP70s (cpHSP70s) (Pulido et al. 2013). These Hsp70-bound DXS proteins can be routed either to the ClpC1 protease for degradation in a housekeeping pathway or refolded by the disaggregase ClpB3 under stress conditions such as heat (Pulido et al. 2016). This DnaJ–HSP70–Clp network forms a central hub of chloroplast proteostasis, preventing the accumulation of toxic protein aggregates and maintaining the functional integrity of plastid enzymes and complexes. Other plastid-localized J-proteins exhibit diverse roles critical for the chloroplast. For example, DJA5 and DJA6 are essential for iron–sulfur cluster biogenesis and plastid morphology (Zhang et al. 2021), while AtJ8, AtJ11, and AtJ20 support photosynthetic efficiency by assisting cpHsp70s in the correct folding and assembly of photosystem components and Rubisco (Portis et al. 2008; Chen et al. 2010). In our barley mutant, disruption of HORVU.MOREX.PROJ.3HG00238870 could compromise the chloroplast's capacity to maintain protein quality control. This may lead to the accumulation of misfolded or damaged proteins, a collapse in the balance between refolding and degradation, and ultimately a breakdown of chloroplast proteostasis and organellar function in affected cells.

Although these two candidates do not appear to interact directly or participate in the same pathway, both may be involved in chloroplast proteostasis. The chloroplast proteome comprises around 3,000 proteins, of which ~90% are encoded in the nucleus and must be imported into the chloroplast. Proteostasis is essential to ensure correct protein folding, assembly, and turnover, thereby maintaining the functionality of photosynthetic and metabolic machinery (Jarvis and López-Juez 2013). While each mutation alone might cause only a mild impairment of proteostasis, their combined effects could disrupt it sufficiently to alter chloroplast morphology and function, which would explain the epistasis we observed. Because the plastid proteome can vary considerably with developmental stage (Teng et al. 2012), these effects might be less pronounced in older plants, potentially accounting for the recovery we observed.

While we previously demonstrated that genetic resequencing can be highly effective for identifying strong candidate genes (Li et al. 2021; Jhingan et al. 2025), our analysis focused only on mutations with a direct effect on the protein sequence of these genes. However, the epistatic interaction between two loci complicates the dissection of the genetic control of this variegation, and regulatory regions—excluded from our initial analysis for feasibility reasons—could also contribute. A finer mapping of each locus in F_3_ populations, in which the counterpart locus is fixed, would help to confirm or refute our candidates, which, in any case, will require functional validation.

## Supplementary Information

Below is the link to the electronic supplementary material.

Supplementary file1 (PDF 196 KB)

Supplementary file2 (XLSX 5852 KB)

## Data Availability

All raw sequencing data produced for this paper is available under projects number PRJEB104329 (GBS data) and PRJEB104330 (WGS data).
