# Supplementary material for: Leaf variegation in a barley EMS mutant is controlled by two epistatic mutations
Source: Theor Appl Genet. 2026 Oct 14;139(11):305. doi: 10.1007/s00122-026-05370-y (PMC13668966; doi:10.1007/s00122-026-05370-y)
Supplement: Supplementary file 1 [file 122_2026_5370_MOESM1_ESM.pdf]

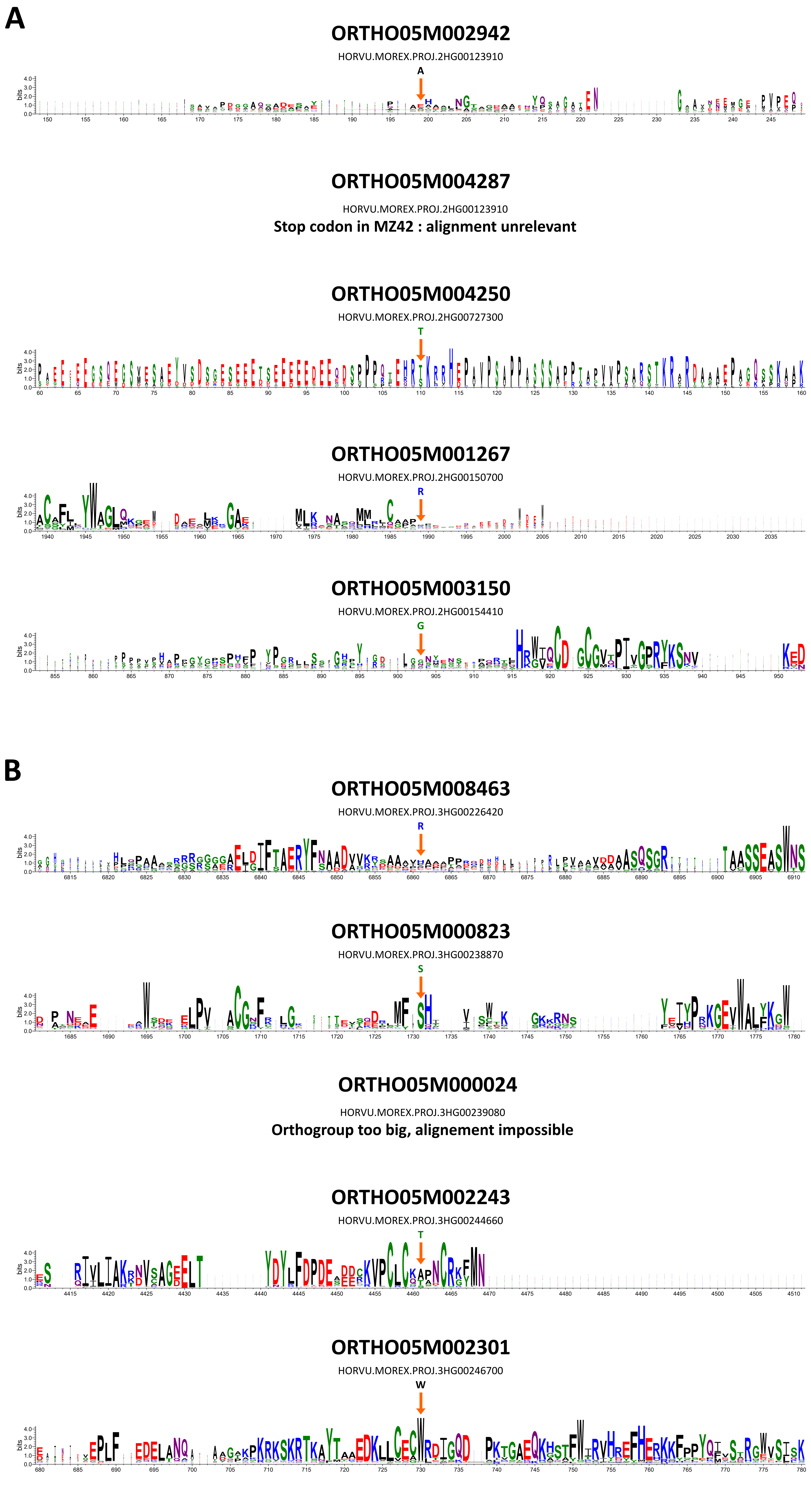

Supplementary Figure 1: Sequence logo of the alignment of the orthogroups of candidate genes for *var4* (A) and *var5* (B) in the 101 bp interval around the polymorphisms carried by the *var4 var5* double mutant. The polymorphism site in the *var4 var5* double mutant is indicated by an orange arrow. The gene ID and the amino acid at the polymorphism site in Morex is specified above the arrow. The orthogroups are defined by PLAZA 5.0 monocots (Van Bel et al. 2022). The sequence logo were generated with WebLogo 3 (Crooks et al. 2004) with the color scheme chemistry.
